# The relationship between emotional regulation and eating behaviour: a multidimensional analysis of obesity psychopathology

**DOI:** 10.1007/s40519-016-0275-7

**Published:** 2016-04-11

**Authors:** Fausta Micanti, Felice Iasevoli, Claudia Cucciniello, Raimondo Costabile, Giuseppe Loiarro, Giuseppe Pecoraro, Fabrizio Pasanisi, GianLuca Rossetti, Diana Galletta

**Affiliations:** 1 Department of Neuroscience, Reproductive Science and Odontostomatology, School of Medicine “Federico II” Naples, Via Pansini, 5, 80122 Naples, Italy; 2 Department of Clinical Medicine and Surgery, Centre Interuniversity Study of Obesity CISRO, School of Medicine “Federico II” Naples, Naples, Italy; 3 Digestive Surgery Unit, School of Medicine Second University of Naples, Naples, Italy

**Keywords:** Obesity, Emotional regulation, Impulsiveness, Body image, Mood, Anxiety, Eating behaviours

## Abstract

**Purpose:**

The aim of this study is to show that the differences among eating behaviours are related to the emotional dysregulation connected to the mental dimensions being part of the obese psychopathology. Eating behaviours can be considered a diagnostic feature at the initial screening for determining the obesity treatment: nutritional or bariatric surgery.

**Methods:**

1828 Obese subjects underwent psychiatric assessment before entering obesity nutritional treatment or bariatric surgery following the multidisciplinary programme. 1121 subjects were selected and enrolled in this study: 850 were inpatients visited or hospitalised at the Obesity Centre or at the Bariatric Surgery Units, 271 were outpatients visited at the Eating Disorder and Obesity Unit. Psychiatric examination was used to exclude psychiatric disorders and investigate eating behaviours distinguished on the basis of food intake rhythm in: gorging, snacking, grazing and binge. They are related to the mental dimensions: impulsiveness, body image, mood and anxiety, taking part in the emotional regulation system. Specific psychometric tools were used to investigate the different mental dimensions of the single eating behaviours and their differences. Statistical analysis of the psychopathological features was performed using ANOVA, ANCOVA, Levene test, Bonferroni’s and Tamhane post hoc test. Significance was set at *p* < 0.05.

**Results:**

Data analysis shows significant differences of psychopathology among all the eating behaviours and an increase in the emotional dysregulation determining maladaptive behaviours.

**Discussion:**

Eating behaviours are connected to the balance of the different features of mental dimensions implicated in the emotional regulation system. They could provide significant clinical information and therefore be part of the obesity diagnostic criteria and therapeutic programme.

## Introduction

Clinical observations of the eating behaviours of obese subjects led to researching the importance of emotions and related mental dimensions in their constitution. Studies about the emotional regulation emphasise that eating is a strategy or an affective response to emotional distress or to a pathological psychological development [1–3]. The emotional eating defined as “a tendency to overeat in response to negative emotions” [4], is connected to pathological personality traits and to a dysregulation of the dysregulated/undercontrolled system particularly for sadness, anxiety, body image and impulsiveness [5, 6]. Impulsiveness, mood, body image and anxiety are the mental dimensions taking part in the emotional regulation and are effective in determining the Eating Behaviour (EB) features in a bi-directional and reciprocal influence [7–12]. Neurobiological studies underline the relation between food reward and exposition to external food cues stressing the importance of external eating in organising food intake rhythm [13–17]. Eating behaviours on the basis of food intake rhythm are distinguished in gorging, snacking, grazing and binge even if there are few investigations about the psychological meaning. The importance of different types of feeding patterns and their potential significance in the pathogenesis and treatment of obesity is largely recognised [1, 3]. Gorging is defined as eating a large amount of food three times a day. It determines a lower increase of Body Mass Index (BMI) than the other types and it is a more positive index for the therapeutic outcome [18]. Snacking is characterised by frequent assumption of snacks in between meals in a person who generally eats at fast foods [19, 20]. Grazing is the repeated “consumption of smaller amounts of food over an extended period of time with an accompanying sense of a lack of control over this eating” [21–24]. Binge is characterised by the loss of control of food intake, mood dysregulation and body shape concern [25, 26].

The aim of this study is to show that the Eating Behaviours can be considered markers of emotional system disorder and are characterised by the differences of quantity and quality of the mental dimensions involved in it. Therefore, they may provide significant clinical information at the initial screening for obesity diagnosis, playing a role in setting up the therapeutic programme.

## Materials and methods

### Recruitment

**Fig. 1:**
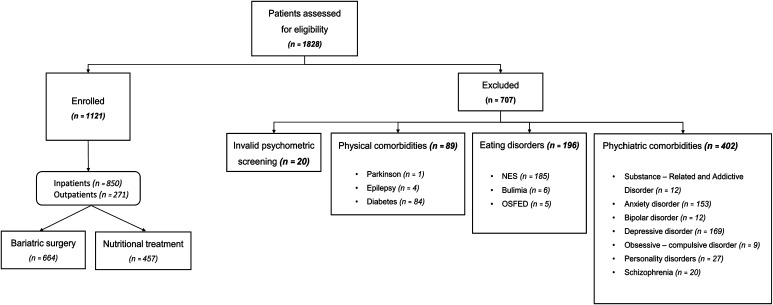
Recruitment flow-chart

From May 2011 to December 2014, 1828 obese subjects asking for nutritional or bariatric surgery treatment underwent psychiatric assessment. The assessment is part of the multidisciplinary programme for obesity treatment of the Interuniversity Centre Study of Obesity, CISRO, School of Medicine “Federico II” Naples. In this study, 1121 subjects were enrolled: 850 inpatients visited or were hospitalised at the Obesity Centre and the Bariatric Surgery Units, 271 outpatients visited at the Eating Disorder and Obesity Unit. Subjects were assessed by a psychiatric evaluation, eating behaviour structured interview according to the cognitive-behavioural model of Garner and Dalle Grave [27] and psychodiagnostic screening. Exclusion criteria were: invalid psychodiagnosis; diabetes because of major incidence of depression; psychiatric disorders determining obesity as consequence of craving or psychopharmacological therapy such as neuroleptics; bipolar disorder because the quality of craving depends on the different psychopathology; anxiety disorders because they can influence the tendency to overeating; eating disorders such as Bulimia Nervosa and Night Eating Syndrome (NES). Bulimia Nervosa was excluded because even if the binge criteria, as DSM-5 stressed, are similar to those of Binge Eating Disorder, the recurrent inappropriate compensatory behaviours suggest a different psychopathological structure. NES was excluded because still now the proposed diagnostic criteria should be better tested according to DSM-5 [25] particularly those of feeding distribution. Moreover, nocturnal eating is considered the result of a dysfunction of circadian rhythm with a dissociation between eating and sleeping rather than the result of a dysfunction of the emotional regulation system [28] (Fig. 1). Subjects were referred to each group considering their prevalent and usual behaviour. Inclusion criterion was binge as symptom of Binge Eating Disorder or of Binge eating disorder of low frequency and/or limited duration” (DSM-5) [25]. Gorging + snacking assessed by psychiatric evaluation were considered as gorging or snacking according to their prevalent frequency. Few episodes of snacking or grazing occurring one time in 3 months associated to gorging were not considered influent in changing gorging assignment. Demographic features were presented in Table 1. All participants signed a written voluntary informed consent form before entering the study.

**Table 1:** Eating behaviour groups

	Gorging pts. 361	Snacking pts. 312	Grazing pts. 261	Binge pts. 187	Total pts. 1,121	*P* value	Post hoc test: significant differences
Sex							
F	207 (57.3 %)	199 (63.8 %)	182 (69.7 %)	155 (82.9 %)	743 (66.3 %)	<0.001^a^	
M	154 (42.7 %)	113 (36.2 %)	79 (30.3 %)	32 (17.1 %)	378 (33.7 %)		
Age, mean (SD)	37.93 (11.69)	36.73 (11.47)	35.72 (11.96)	34.56 (12.03)	36.52 (11.80)	0.009^b^	Binge versus gorging *p* = 0.009^c^
BMI, mean (SD)	46.20 (8.66)	45.72 (8.53)	45.51 (8.21)	44.93 (8.56)	45.69 (8.50)	0.403^b^	

Demographic characteristics

^a^ Chi square test, ^b^ ANOVA, ^c^ Bonferroni

## Methods

Psychiatric assessment consisted of:

1. Psychiatric examination to exclude psychiatric disorders according to the Diagnostic and Statistical Manual of Mental Disorders-5 [25] (Fig. 1).
2. A structured interview to identify Eating Behaviour types.
3. Psychometric evaluation performed by rating scales validated for the psychopathological dimensions to be investigated: Binge Eating Scale (BES) [29, 30], Barratt Impulsiveness Scale (BIS-11) for impulsiveness [31–35]; Eating Disorder Inventory (EDI-2) for eating behaviour [36, 37]; Body Uneasiness Test (BUT) for body image dissatisfaction and uneasiness [38, 39]; Beck Depression Inventory-II (BDI-II) for mood [40, 41]; State-Trait Anxiety Inventory (STAI-Y) for anxiety [42, 43]; Short Form Health Survey (SF-36) for quality of life [44, 45].

Impulsiveness was evaluated using global scores of BES and BIS-11 and its inner factors: cognitive impulsiveness (CF), motor impulsiveness (MF) and non planning impulsiveness (NpF) and the EDI-2 subscales: Bulimia (Bu) and Impulse Regulation (IR), since the results were significantly different among eating behaviour groups at multiple comparisons. The EDI-2 subscales were chosen since Bulimia explores the tendency to think or act out binge episodes; the Impulse Regulation measures the ability to regulate impulsive behaviour, especially binge and the tendency to impulse reaction.

Body image dimension was evaluated using BUT A excluding the Depersonalization (D) factor because it specifically estimates the interference of mood on body image, which is the object of a distinct assessment herein. The EDI-II subscale Body Dissatisfaction (BD) was chosen because it indicates the mental condition of not being satisfied with one’s own physical appearance.

Mood dimension was evaluated using the global scores of the BDI-II; the BUT A factor Depersonalization that measures the tendency to depression related to body uneasiness and dissatisfaction; the EDI-2 subscale Ineffectiveness (I) which assesses feelings of inadequacy, insecurity, worthlessness and having no control over one’s own life and the SF-36 Mental Component Summary (MCS) to investigate the relation between mental health, mood and quality of life.

Anxiety was evaluated using the STAI-Y global score which measures trait anxiety. This study met the criteria of a cross-sectional design.

### Statistics

The Chi square test was used to assess the homogeneity of gender distribution among the groups. The Analysis of variance (ANOVA) was performed to analyze the differences in mean values on rating scales scores in the four groups of obese subjects with the different eating behaviours. Levene test was used to assess the equality of variances. Bonferroni post hoc test was used for multiple comparisons. The analysis of covariance (ANCOVA) in a univariate general linear model was performed to test the interaction of age and gender scores as covariates. In all tests, significance was set at *p* < 0.05 (two-tailed).

All analyses were carried out using IBM^®^ SPSS^®^ Statistics version 20.

## Results

Demographics of the sample are reported in Table 1.

Gender distribution among the groups was significantly different, with a progressive increment in female percentage from gorging to binge (Chi square test, *p* < 0.001). Mean age was significantly different among subjects groups (ANOVA, *p* = 0.009). At the post hoc test, binge subjects had significantly lower mean age compared to gorging ones (Bonferroni, *p* = 0.009). No other significant differences in mean age among groups were found.

Mean scores with standard deviations among the four EB types for each psychopathological dimension studied are reported in Table 2.

**Table 2:** Mean scores with standard deviations among the four EB for every mental dimension

	Gorging pts. 361	Snacking pts. 312	Grazing pts. 261	Binge pts. 187	Total pts. 1121	*P* value	Post hoc test: significant differences
	Mean (SD)	Mean (SD)	Mean (SD)	Mean (SD)	Mean (SD)		
Impulsiveness							
BES	8.00 (5.68)	13.60 (5.81)	19.72 (6.40)	28.37 (5.77)	15.68 (9.26)	<0.001^a^	Binge versus other EB: *p* < 0.001^b^
							Grazing versus other EB: *p* < 0.001^b^
							Snacking versus other EB: *p* < 0.001^b^
EDI-2 (Bu)	1.56 (2.22)	2.97 (3.15)	4.81 (3.87)	9.18 (4.75)	3.98 (4.29)	<0.001^a^	Binge versus other EB: *p* < 0.001^b^
							Grazing versus other EB: *p* < 0.001^b^
							Snacking versus other EB: *p* < 0.001^b^
EDI-2 (IR)	2.54 (3.59)	3.66 (4.06)	5.44 (5.00)	10.64 (6.81)	4.88 (5.48)	<0.001^a^	Binge versus other EB: *p* < 0.001^b^
							Grazing versus other EB: *p* < 0.001^b^
							Snacking versus gorging: *p* = 0.013^b^
BIS-11 (Ba)*	56.56 (9.32)	61.87 (9.06)	66.36 (9.42)	76.40 (10.36)	63.63 (11.62)	<0.001^a^	Binge versus other EB: *p* < 0.001^b^
							Grazing versus other EB: *p* < 0.001^b^
							Snacking versus other EB: *p* < 0.001^b^
*Subscales							
BIS-11 (CF)	13.36 (3.12)	14.59 (3.21)	16.13 (3.52)	19.05 (4.32)	15.30 (3.98)	<0.001^a^	Binge versus other EB: *p* < 0.001^b^
							Grazing versus other EB: *p* < 0.001^b^
							Snacking versus other EB: *p* < 0.001^b^
BIS-11 (MF)	19.38 (4.03)	20.99 (4.02)	22.12 (4.36)	25.68 (4.94)	21.52 (4.77)	<0.001^a^	Binge versus other EB: *p* < 0.001^b^
							Grazing versus snacking: *p* = 0.01^b^
							Grazing versus gorging: *p* < 0.001^b^
							Snacking versus gorging: *p* < 0.001^b^
BIS-11 (NPF)	23.85 (4.72)	26.53 (4.38)	28.11 (4.58)	31.71 (4.46)	26.90 (5.28)	<0.001^a^	Binge versus other EB: *p* < 0.001^b^
							Grazing versus other EB: *p* < 0.001^b^
							Snacking versus other EB: *p* < 0.001^b^
Body image							
EDI-2 (BD)	14.20 (6.56)	16.52 (6.00)	18.06 (6.47)	19.86 (5.56) 16.69 (6.54)		<0.001^a^	Binge versus grazing: *p* = 0.016^b^
							Binge versus snacking: *p* < 0.001^b^
							Binge versus gorging: *p* < 0.001^b^
							Grazing versus snacking: *p* = 0.019^b^
							Grazing versus gorging: *p* < 0.001^b^
							Snacking versus gorging: *p* < 0.001^b^
BUT (WP)	1.97 (1.30)	2.46 (1.22)	2.90 (1.18)	3.58 (1.34)	2.59 (1.37)	<0.001^a^	Binge versus other EB: *p* < 0.001^b^
							Grazing versus other EB: *p* < 0.001^b^
							Snacking versus other EB: *p* < 0.001^b^
BUT (BIC)	2.12 (1.28)	2.75 (1.38)	3.14 (1.19)	3.86 (1.76)	2.82 (1.51)	<0.001^a^	Binge versus other EB: *p* < 0.001^b^
							Grazing versus snacking: *p* = 0.005^b^
							Grazing versus gorging: *p* < 0.001^b^
							Snacking versus gorging: *p* < 0.001^b^
BUT (A)	0.94 (1.06)	1.44 (1.34)	1.92 (1.26)	2.81 (1.51)	1.62 (1.42)	<0.001^a^	Binge versus other EB: *p* < 0.001^b^
							Grazing versus other EB: *p* < 0.001^b^
							Snacking versus other EB: *p* < 0.001^b^
BUT (CSM)	0.92 (0.88)	1.34 (0.97)	1.56 (0.95)	2.12 (1.02)	1.39 (1.03)	<0.001^a^	Binge versus other EB: *p* < 0.001^b^
							Grazing versus snacking: *p* = 0.03^b^
							Grazing versus gorging: *p* < 0.001^b^
							Snacking versus gorging: *p* < 0.001^b^
Depression							
EDI-2 (GSI)	1.48 (1.00)	2.04 (1.22)	2.44 (1.02)	3.17 (1.22)	2.14 (1.25)	<0.001^a^	Binge versus other EB: *p* < 0.001^b^
							Grazing versus other EB: *p* < 0.001^b^
							Snacking versus other EB: *p* < 0.001^b^
EDI-2 (I)	3.98 (4.25)	5.81 (5.37)	7.80 (5.55)	12.93 (6.56)	6.87 (6.12)	<0.001^a^	Binge versus other EB: *p* < 0.001^b^
							Grazing versus other EB: *p* < 0.001^b^
							Snacking versus other EB: *p* < 0.001^b^
BUT (D)	0.95 (1.13)	1.47 (1.28)	2.02 (1.36)	2.89 (1.44)	1.66 (1.45)	<0.001^a^	Binge versus other EB: *p* < 0.001^b^
							Grazing versus other EB: *p* < 0.001^b^
							Snacking versus other EB: *p* < 0.001^b^
BDI-II (G)	10.48 (7.66)	13.44 (8.62)	17.86 (10.61)	26.39 (9.84)	15.67 (10.59)	<0.001^a^	Binge versus other EB: *p* < 0.001^b^
							Grazing versus other EB: *p* < 0.001^b^
							Snacking versus other EB: *p* < 0.001^b^
SF36 (MCS)	70.64 (19.81)	62.19 (20.56)	55.59 (20.91)	39.13 (21.64)	59.55 (23.18)	<0.001^a^	Binge versus other EB: *p* < 0.001^b^
							Grazing versus snacking: *p* = 0.001^b^
							Grazing versus gorging: *p* < 0.001^b^
							Snacking versus gorging: *p* < 0.001^b^
Anxiety							
STAI-Y	37.06 (10.05)	40.84 (10.53)	46.80 (12.54)	55.96 (11.59)	43.53 (12.88)	<0.001^a^	Binge versus other EB: *p* < 0.001^b^
							Grazing versus other EB: *p* < 0.001^b^
							Snacking versus other EB: *p* < 0.001^b^

Ba, barratt; Bu, bulimia; IR, impulse regulation, BD, body dissatisfaction; WP, weight phobia; BIC, body image concern; A, avoidance; CSM, check self monitoring; GSI, global symptom index, I, ineffectiveness; D, depersonalization; G, global; SF36 MCS, mental component summary

^a^ ANOVA, ^b^ Bonferroni

**Fig. 2:**
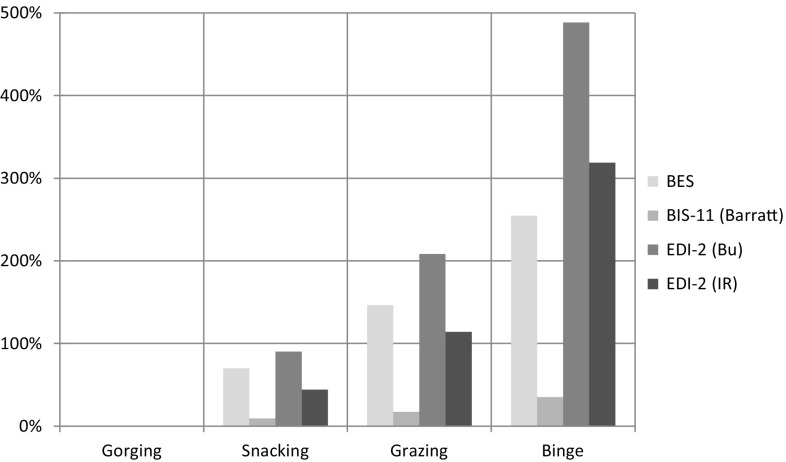
*Impulsiveness*. *Bar chart* detailing the percentage increase of each mean value (Bu, bulimia; IR, impulse regulation) from minimum (Gorging) to maximum (Binge); e.g. Binge has EDI-2 Bulimia mean value increased approximately +500 % (six times) compared with Gorging

At Impulsiveness assessment, significant differences among the four EB types were found on the mean scores of BES, BIS-11, EDI-2 Bu and IR subscales (ANOVA, *p* < 0.001 for each one). In all these assessments, mean scores were significantly higher in binge compared to all other eating behaviour types (Bonferroni, *p* < 0.001 in all assessments); in grazing compared to snacking and gorging (Bonferroni, *p* < 0.001 in all assessments); in snacking compared to gorging (BES, BIS-11, EDI-2 Bulimia subscale Bonferroni, *p* < 0.001; EDI-2 Impulsiveness subscale Bonferroni, *p* = 0.013). The progressive increase in mean scores from gorging to binge is shown in Fig. 2.

**Fig. 3:**
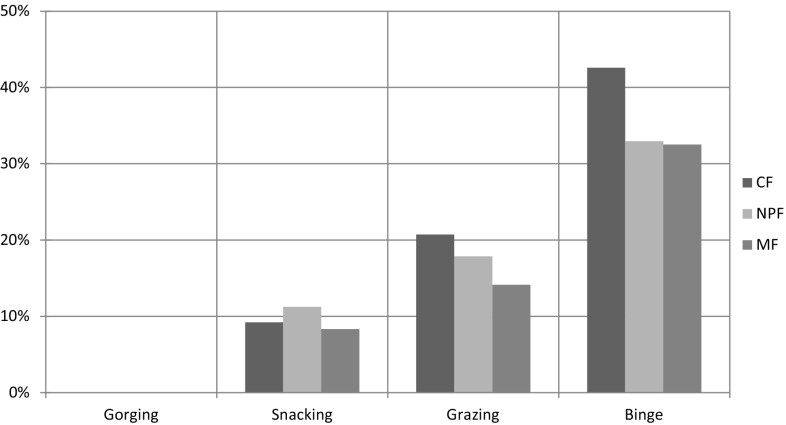
*Impulsiveness.* *Bar chart* detailing the percentage increase of each factor mean value from minimum (Gorging) to maximum (Binge); e.g. Binge has cognitive factor (CF) mean value increased approximately +43 % (0.4 times) compared with Gorging

Significant differences among the four eating behaviours were found at CF, MF, NpF, factors of BIS-11. (ANOVA, *p* < 0.001 for each factor). Mean scores were significantly higher in binge compared to all the others (Bonferroni, *p* < 0.001 in all assessments); in grazing compared to snacking and gorging (MF Bonferroni, *p* = 0.01 vs. snacking; in all other assessments Bonferroni, *p* < 0.001); and in snacking compared to gorging (Bonferroni, *p* < 0.001 in all assessments). Significant differences among the four eating behaviours were also found at the other investigated mental dimensions (Fig. 3).

1. Body Image, i.e, the EDI-2 BD subscale and the BUT factors WP, BIC, A, CSM, GSI (ANOVA, *p* < 0.001 for each factor). As above, mean scores were significantly higher in binge compared to all others eating behaviours (BD EDI-2 subscale Bonferroni, *p* = 0.016 vs. grazing; in all other assessments Bonferroni, *p* < 0.001); in grazing compared to snacking and gorging (BD EDI-2 Bonferroni, *p* = 0.019 vs. snacking; BUT A BIC Factor Bonferroni, *p* = 0.005 vs. snacking; BUTA CSM factor Bonferroni, *p* = 0.03 vs. snacking; in all other assessments Bonferroni, *p* < 0.001); and in snacking compared to gorging (Bonferroni, *p* < 0.001 in all assessments) (Fig. 4).

**Fig. 4:**
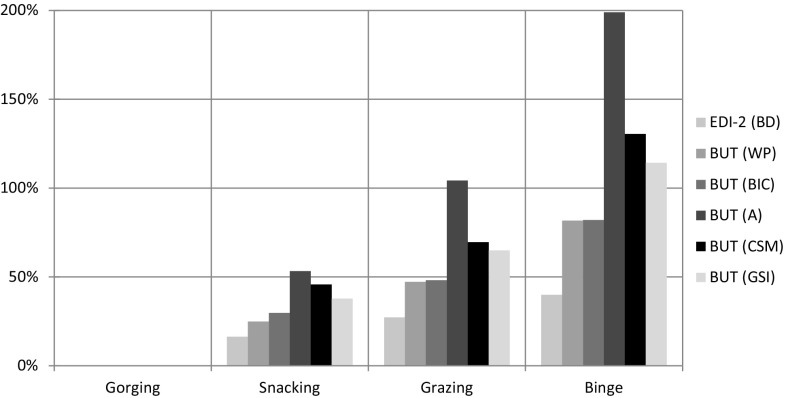
*Body image*.* Bar chart* detailing the percentage increase of each mean value (*A* avoidance, *CSM* check self monitoring, *GSI* global symptom index, *BIC* body image concern, *WP* weight phobia, *EDI-2BD* body dissatisfaction) from minimum (Gorging) to maximum (Binge); e.g. Binge has BUT Avoidance mean value increased approximately +200 % (three times) compared with Gorging
2. Mood, i.e, the EDI-2 IN subscale; BUT D Factor; BDI-II global score; and the SF-36 MCS (ANOVA, *p* < 0.001 for each one). IN and D subscales and BDI-II mean scores were significantly higher in binge compared to all other EB types (Bonferroni, *p* < 0.001 in all assessments); in grazing compared to snacking and gorging (Bonferroni, *p* < 0.001 in all assessments); and in snacking compared to gorging (Bonferroni, *p* < 0.001 in all assessments). The MCS mean score was significantly higher in gorging compared to all other Eating behaviour types (Bonferroni, *p* < 0.001 in all assessments); in snacking compared to grazing and binge (Bonferroni, *p* < 0.001 in all assessments); and in grazing compared to binge (Bonferroni, *p* < 0.001) (Fig. 5).

**Fig. 5:**
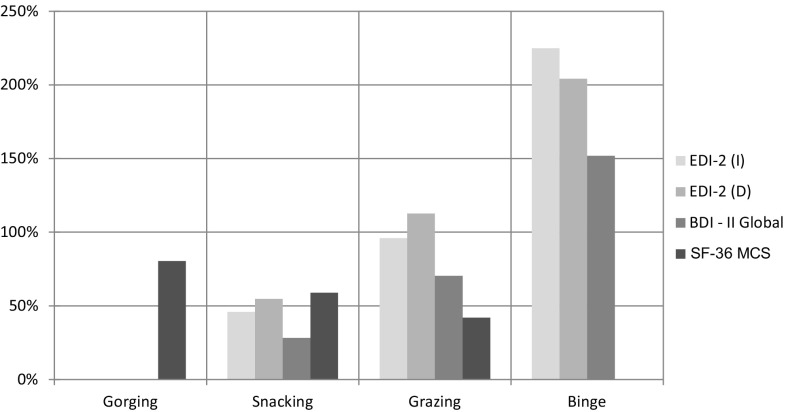
*Mood.* *Bar chart* detailing the percentage increase of EDI-2 subscales mean value (*IN* ineffectiveness, *D* depersonalization); of SF-36 MCS (Mental Component Summary) from minimum (Gorging) to maximum (Binge), *except for SF36 MCS *bar chart* decreasing from maximum to minimum. E.g. Binge has EDI-2 Ineffectiveness mean value increased approximately +230 % (3.3 times) compared with Gorging
3. Anxiety (ANOVA, *p* < 0.001): STAI-Y mean score was significantly higher in binge compared to all other EB types (Bonferroni, *p* < 0.001 in all assessments); in grazing compared to snacking and gorging (Bonferroni, *p* < 0.001 in all assessments); and in snacking compared to gorging (Bonferroni, *p* < 0.001) (Fig. 6).

**Fig. 6:**
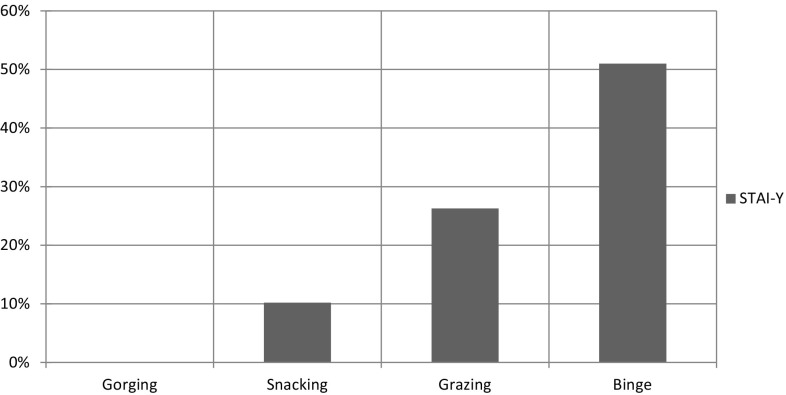
*Anxiety*. *Bar chart* detailing the percentage increase of STAI-Y mean value from minimum (Gorging) to maximum (Binge); Binge has global STAI-Y mean value increased approximately +50 % (0.5 times) compared with Gorging

Since mean age and gender distribution were significantly different among the four eating behaviours, the analysis of covariance (ANCOVA) was carried out in a univariate general linear model to establish whether the significant differences in psychopathology assessment described above may also be affected by these two variables. Results are reported in Table 2. Partial Eta squared scores are also reported as estimates of effect size.

Age significantly covaried with the BIS-11 CF only. On the other hand, gender covaried with several psychopathology measures, i.e. BUT A factors (ANCOVA, *p* = 0.005); BUT A CSM (ANCOVA, *p* = 0.027); EDI-2 IN subscale (ANCOVA, *p* = 0.046). In all these cases, the female gender significantly increased the propensity to have higher scores.

## Discussion

Data analysis highlights that the mutual interference of impulsiveness, body image, mood and anxiety contribute to diversifying Eating Behaviours. The increase of BUT scores of the female gender is consistent with clinical observations that body dissatisfaction and uneasiness are stronger in females than in males and are the most powerful stimulus to undergo obesity treatment.

### Impulsiveness

Examining the impulsiveness data (Fig. 2), BES and BIS-11 mean scores show a significant statistical increase of the impulse response from gorging to binge. Many studies emphasiseemphasise the relationship between impulsiveness and overeating, particularly the link with external eating. High scores of impulsiveness are more sensible in obese woman as Ancova gender (Table 3) elucidates and associated with both cognitive and motor factors of impulsiveness. These data (Table 2) agree with those stressing the differences in eating response to food cues [46], with a personal trait related to the single impulsive disorder and with the individual response to inner factors such as the sensation of hunger. The analysis of the internal factors of BIS-11 emphasises that the organisation of the impulse response is influenced by the cognitive factor and that the different Eating Behaviours have a progressive increase in the same factor scores up to 43 % of binge underlining the progressive dysregulation of the impulse control. Data results (Fig. 2) highlight that gorging has low scores of the three factors and of the related EDI-2 subscales stressing that overweight is due to external eating style (tradition, palatable habits, hunger) more than to the internal hunger sensation or to the prevalence of rash-spontaneous impulsiveness (NpF). Snacking also shows a little increase of values nearing those of gorging. Therefore, gorging and snacking can be considered to be associated with a better organisation of food impulse. This organisation becomes worse from gorging to binge (Fig. 2). Grazing presents an increase in the CF and MF factors but not in the NpF. These data are consistent with the hypothesis that motor impulsiveness correlates much more with external eating rather than with the response to internal cues such as craving [46, 47]. They suggest why obese subjects with grazing behaviour have a great consumption of unhealthy food in a determined period of time and have the sensation of loss of control, but really they do not lose it. Binge is characterised by high scores of the three factors, but data analysis does not indicate a prevalence of NpF, on the contrary there is an internal prevalence of CF (Table 2). This result is unexpected even if the high scores of MF and NpF suggest that every component of impulsiveness is dysregulated. The CF increase could explain the cognitive impulse of finding, buying or cooking food that comes before or during a binge episode. Further studies about the inner factors of impulsiveness related to food reward could clarify the nature of craving. The result of the relationship between age and CF factor (Table 3) can be explained by the worsening of the cognitive function (Problem Solving and Social Cognition tasks) related to age, but further studies must be applied considering the global cognitive impairment of obese subjects.

**Table 3:** Interaction of age and gender on mean scores of each psychopathological dimension among the four groups

	EB * age interaction		EB * gender interaction	
	Significance^a^	Partial Eta squared	Significance^a^	Partial Eta squared
BES	*p* = 0.892	0.117	*p* = 0.158	0.005
BIS-11 (Ba)	*p* = 0.149	0.005	*p* = 0.348	0.003
EDI-2 (Bu)	*p* = 0.480	0.002	*p* = 0.175	0.004
EDI-2 (IR)	*p* = 0.973	<0.001	*p* = 0.284	0.003
CF	*p* = 0.023^b^	0.009	*p* = 0.861	0.001
FpN	*p* = 0.784	0.001	*p* = 0.207	0.004
MF	*p* = 0.148	0.005	*p* = 0.263	0.004
EDI-2 (BD)	*p* = 0.421	0.003	*p* = 0.945	<0.001
BUT (WP)	*p* = 0.409	0.003	*p* = 0.086	0.006
BUT (BIC)	*p* = 0.596	0.002	*p* = 0.632	0.002
BUT (A)	*p* = 0.286	0.003	*p* = 0.005^b^	0.012
BUT (CSM)	*p* = 0.543	0.002	*p* = 0.027^b^	0.008
BUT (GSI)	*p* = 0.186	0.004	*p* = 0.075	0.006
EDI-2 (I)	*p* = 0.288	0.003	*p* = 0.046^b^	0.007
BUT (D)	*p* = 0.959	<0.001	*p* = 0.088	0.006
BDI-II (G)	*p* = 0.878	0.001	*p* = 0.276	0.003
SF36 (ISM)	*p* = 0.904	0.001	*p* = 0.894	0.001
STAI-Y	*p* = 0.973	<0.001	*p* = 0.848	0.001

^a^ Analysis of covariance (ANCOVA); ^b^ significant interaction

### Body image

Body image dissatisfaction is considered one of the most powerful stimuli for controlled or uncontrolled food intake [48]. The cognitive–emotional and affective components of Body image are effective in inducing dietary restraint, overeating and maladaptive food patterns. The importance of emotional dysregulation on body image structure is well known even if its relationship has not been fully explored [49, 50]. Sufficient cognitive function can help obese people to cope with obesity treatment; on the contrary the prevalence of the disorder of the affective component can be deemed a negative predictor of the outcome [51]. Studies show that Body checking, explored with BUT A factor Check Self Monitoring regulates emotions negatively by confirming the subject’s shape fears. Avoidance, also checked by BUT A factor Avoidance, is related to the failure of escaping the pressure of temporary emotional dysregulation and is significantly associated with binge [52]. Data analysis of eating behaviours (Fig. 4) highlight that gorging and snacking have low scores of the Global Distress Index, of all BUT A factors and EDI-2 Body Dissatisfaction subscale. These data emphasiseemphasise that the relationship between emotional regulation and body image produces a negative body image but the low scores of Avoidance and Check Self Monitoring indicate that patients are able to cope with the feeling of shame and low self-esteem determining negative body image. Moreover, these data are related to the low scores of impulsiveness, depression and anxiety indicating a stronger ability to cope with emotion determining a reinforcement of motivation to obesity treatment. Grazing (Fig. 4) shows a general increase of body uneasiness and dissatisfaction demonstrated by the consistent increase (more than 50 %) of the mean values of Check Self Monitoring and Avoidance stressing that control of the negative emotions is dysregulated. These data and the increase of the mean values of the Motor impulsiveness and non planning impulsiveness factors underline the difficulty in organising emotional control under inner and external cues. Binge shows an increase of all BUT factor scores and EDI-2 Body Dissatisfaction subscale (until 200 %) that associated with the prevalence of NpF and MF impulsiveness factors (Fig. 4) highlight the inability to control body shape concern and give any organised response to external and inner pressure. They are consistent with the analysis of the emotional regulation and body image in binge subjects [51, 53, 54] and can be an answer to the recurring clinical observation of their lack of worry about getting fat.

### Mood and anxiety

The data analysis shows the increase of BDI score, of the Depersonalization factor and Ineffectiveness subscale from gorging to binge. The relationship between mood and obesity includes the emotional regulation, activation of the brain region of reward with differences between palatable food and high fat food. Gorging and snacking have a low level of mood score related to the metabolic syndrome that influences mood negatively in a complex way including activation of the food reward, the dopaminergic axis and the choice of food, altered cortisol level and high insulin resistance in a bi-directional way [55–57]. Grazing presents an increase of 100 % (Fig. 5) of Depersonalization and Ineffectiveness scores pointing out that the sense of estrangement and the tendency to feel inadequate interfere with the ability to control the emotional regulation. The increase of the SF-36 Mental Component Summary (MCS) emphasises a worsening of the relationship between mental health and Eating Behaviour. Binge is characterised by high scores particularly by the increase of 200 % in Depersonalization and Ineffectiveness scores (Fig. 5). The MCS increase of 100 % underlines that mental health interferes strongly with the emotional regulation. Binge as symptom of Binge Eating Disorder, according to the study analysis, is associated with a global dysregulation of emotional regulation system.

Grazing and binge show an increase in the anxiety score (Fig. 6), but the score in grazing is lower than in binge clarifying the anxious sensation of losing control without losing it. Moreover, the lack of loss of control in grazing could be determined by the lower levels of MF and NpF impulsiveness factors than in binge. On the contrary high levels of anxiety can explain the arousal that binge individuals feel before the acting out and the loss of control of food intake.

## Conclusions

The overall analysis of the data highlights the differences in eating behaviours and the characteristics that could contribute to success or failure of obesity treatment. It is consistent with literature data emphasising the necessity of flexible models for obesity treatment. The nutritional or bariatric surgery outcome can be improved by treatment focusing on the emotional regulation such as psychotherapy, life style change and also psychopharmacological treatment [58–63].

Limitations of the present study is the lack of considering samples of the eating behaviours in subjects suffering from overweight for a short time in order to understand if the persistence of obesity can influence the characteristics of the mental dimensions above all body image and mood. Moreover, this study does not present the results at the loss and weight maintenance period of psychological treatment before diet or bariatric surgery based on Eating Behaviour distinction.

Eating behaviours can be considered markers of obesity psychopathology and could contribute to the diagnosis performed by the multidisciplinary team at the initial screening and the subsequent treatment.
